# Fusion of the C-terminal triskaidecapeptide of hirudin variant 3 to alpha1-proteinase inhibitor M358R increases the serpin-mediated rate of thrombin inhibition

**DOI:** 10.1186/1471-2091-14-31

**Published:** 2013-11-11

**Authors:** Leigh Ann Roddick, Varsha Bhakta, William P Sheffield

**Affiliations:** 1 Pathology and Molecular Medicine, McMaster University, 1280 Main Street West, Hamilton, ON L8S 4 K1, Canada; 2 Research and Development, Canadian Blood Services, HSC 4N66 McMaster University, 1280 Main Street West, Hamilton, ON L8S 4 K1, Canada

**Keywords:** Alpha1-proteinase inhibitor, Thrombin, Hirudin, Serpins, Coagulation, Inhibition

## Abstract

**Background:**

Alpha-1 proteinase inhibitor (API) is a plasma serpin superfamily member that inhibits neutrophil elastase; variant API M358R inhibits thrombin and activated protein C (APC). Fusing residues 1-75 of another serpin, heparin cofactor II (HCII), to API M358R (in HAPI M358R) was previously shown to accelerate thrombin inhibition over API M358R by conferring thrombin exosite 1 binding properties. We hypothesized that replacing HCII 1-75 region with the 13 C-terminal residues (triskaidecapeptide) of hirudin variant 3 (HV3_54-66_) would further enhance the inhibitory potency of API M358R fusion proteins. We therefore expressed HV3API M358R (HV3_54-66_ fused to API M358R) and HV3API RCL5 (HV3_54-66_ fused to API F352A/L353V/E354V/A355I/I356A/I460L/M358R) API M358R) as N-terminally hexahistidine-tagged polypeptides in *E. coli.*

**Results:**

HV3API M358R inhibited thrombin 3.3-fold more rapidly than API M358R; for HV3API RCL5 the rate enhancement was 1.9-fold versus API RCL5; neither protein inhibited thrombin as rapidly as HAPI M358R. While the thrombin/Activated Protein C rate constant ratio was 77-fold higher for HV3API RCL5 than for HV3API M358R, most of the increased specificity derived from the API F352A/L353V/E354V/A355I/I356A/I460L API RCL 5 mutations, since API RCL5 remained 3-fold more specific than HV3API RCL5. An HV3 54-66 peptide doubled the Thrombin Clotting Time (TCT) and halved the binding of thrombin to immobilized HCII 1-75 at lower concentrations than free HCII 1-75. HV3API RCL5 bound active site-inhibited FPR-chloromethyl ketone-thrombin more effectively than HAPI RCL5. Transferring the position of the fused HV3 triskaidecapeptide to the C-terminus of API M358R decreased the rate of thrombin inhibition relative to that mediated by HV3API M358R by 11-to 14-fold.

**Conclusions:**

Fusing the C-terminal triskaidecapeptide of HV3 to API M358R-containing serpins significantly increased their effectiveness as thrombin inhibitors, but the enhancement was less than that seen in HCII 1-75–API M358R fusion proteins. HCII 1-75 was a superior fusion partner, in spite of the greater affinity of the HV3 triskaidecapeptide, manifested both in isolated and API-fused form, for thrombin exosite 1. Our results suggest that HCII 1-75 binds thrombin exosite 1 and orients the attached serpin scaffold for more efficient interaction with the active site of thrombin than the HV3 triskaidecapeptide.

## Background

Alpha-1-proteinase inhibitor (API) belongs to the serpin superfamily, a class of proteins whose members typically form inhibitory complexes with the serine proteinases they regulate [1-4]. API is the most abundant serpin in mammalian plasma, circulating at a concentration of 20-50 μM [5]. API protects tissues from attack by inflammatory proteinases; notably, in alveolar fluid it protects lung parenchymal cells and elastin from destruction by neutrophil elastase [6]. This physiological role is underscored by the increased risk of emphysema manifested by individuals with hereditary deficiency of API [6]. API also inhibits other serine proteinases such as thrombin [7], coagulation factor XIa [8], and activated protein C (APC) [9], but at rates at least six orders of magnitude less rapid than its inhibition of elastase; it also efficiently inhibits trypsin, but would not be expected to come in contact with this digestive system enzyme under physiological conditions.

Serpins inhibit their cognate proteinases via a complex mechanism initiated when the reactive centre loop (RCL) surface structure forms an encounter complex with the active site of a protease [2]. Attack of the protease on the reactive centre scissile bond of the RCL triggers the release of stored energy [10], a rapid insertion of the RCL into an underlying β-sheet [11], and a major translocation of the protease [12], still attached to the RCL as a covalently bound acyl intermediate [13], to the opposite pole of the serpin. The rapid translocation distorts the active site of the protease, preventing completion of the catalytic cycle [14,15], and rendering the serpin inhibitory complex physiologically irreversible. Crystal structures of API in intact form [16], in RCL-cleaved form [17], in encounter complexes with trypsin [18], and in covalent serpin-enzyme complexes with trypsin [15] and elastase [14] have contributed to the elucidation of this mechanism.

The naturally occurring Pittsburgh mutation, of the scissile bond in API from M358-S359 to R358-S359, was discovered in a patient with an ultimately fatal bleeding tendency [19], although two other unrelated individuals with the same mutation and clinically milder consequences have since been reported [20,21]. Mutant API M358R was found to inhibit thrombin [19,22], factor XIa [22], and APC [23] from 5,000-to 8,000-fold more rapidly than its wild-type counterpart. Our group [24,25] and others [26,27] have reported that introducing additional mutations into the RCL of API M358R enhanced its specificity as a thrombin inhibitor, primarily by decreasing its potency as an APC inhibitor. Noting that the 75 amino acid N-terminal acidic extension of heparin cofactor II (HCII), a thrombin-specific inhibitory serpin, had no counterpart in API [1], we fused HCII 1-75 to API M358R, and demonstrated a > 5-fold increase in the rate of thrombin inhibition [24,28], and a superior ability to limit thrombus size in murine arterial and venous thrombosis models, relative to API M358R [29].

The acidic extension of HCII accelerates thrombin inhibition by binding to thrombin exosite 1, a cluster of charged residues involved in binding thrombin co-factors such as thrombomodulin and substrates such as fibrinogen and coagulation factor V [30]. This deduction is supported by: a partially resolved X-ray crystal structure of the HCII-S195A thrombin structure [31]; reduced rates of inhibition of HCII and HCII fusion proteins on exosite-1-disrupted forms of thrombin [32-35]; and direct binding studies of either HCII 1-75 or its smaller derivatives [36,37]. The leech anticoagulant protein hirudin is the most potent known polypeptide inhibitor of thrombin [38], and owes some of its high affinity for thrombin to the binding of its 11-13 C-terminal residues, depending on the isoform [39], to thrombin exosite 1. We [36] and others [37] have demonstrated that hirudin C-terminal peptides bind more tightly to thrombin than HCII 1-75 or its derivatives. Accordingly, in the current study we tested the primary hypothesis that fusion of the C-terminal 13 amino acids of hirudin variant 3 (HV3), the most potent hirudin isoform [39], to API M358R, would increase the rate of thrombin inhibition of API M358R by conferring on this mutant serpin the ability to bind thrombin exosite 1. We also addressed our secondary hypothesis, that an HV3 fusion to API M358R would inhibit thrombin more effectively than the analogous HCII-API fusion protein.

## Methods

### Peptides

**Figure 1:**
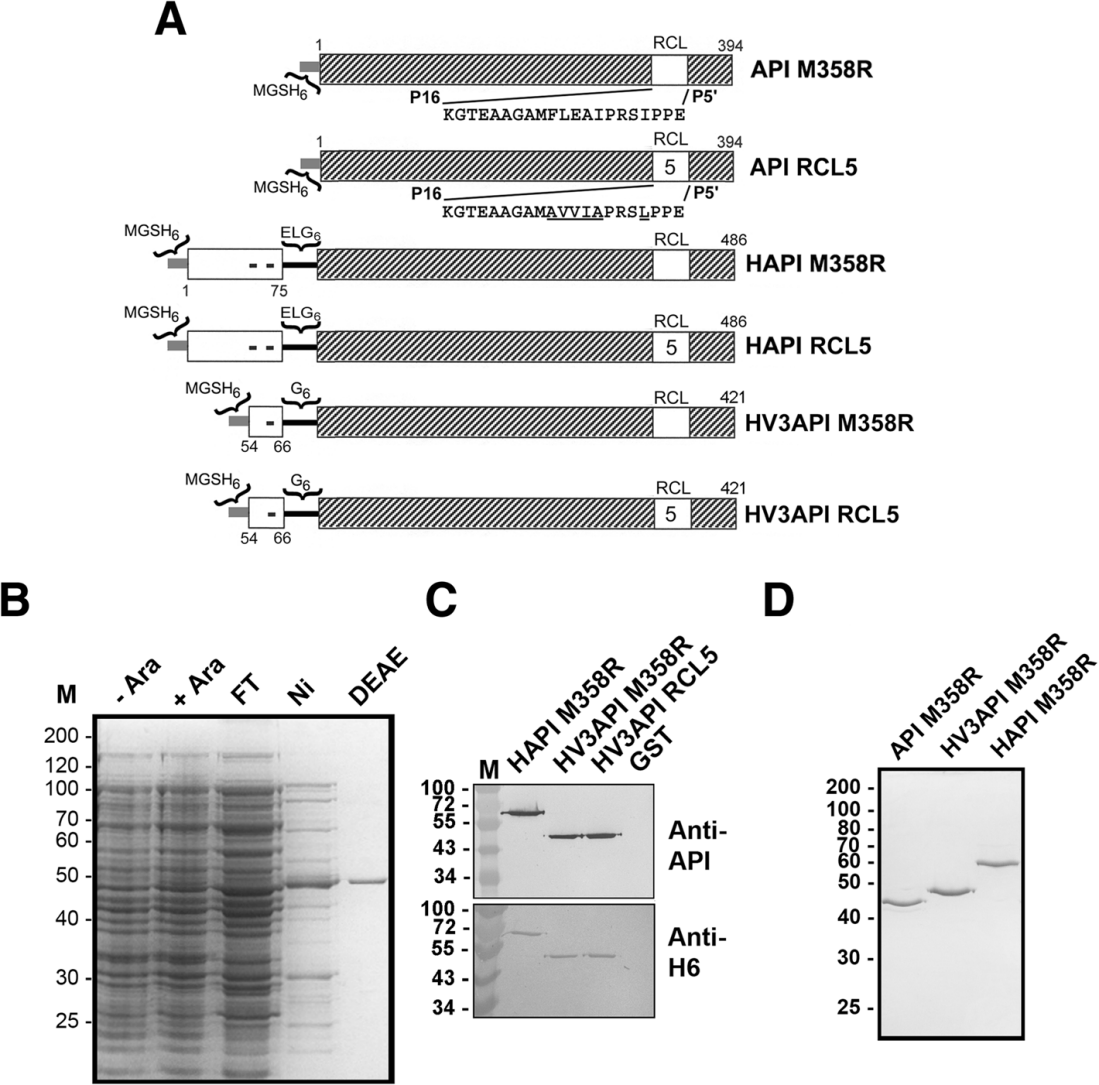
**Schematic and electrophoretic representation of proteins and peptides employed.** Polypeptides comprising portions of API M358R (hatched bar, with residue numbers above) with or without N-terminal extensions (white) derived from HCII (symbols inset, and residue number below) or HV3 (symbol inset, and residue numbers below) are represented schematically in **Panel A**. Each protein contains an N-terminal MGSH6 tag (at left of each schematic representation, thin grey bar). Fusion proteins contain a spacer peptide (thin black bar) following the N-terminal extension of either 6 or 8 residues identified above the bar. Reactive centre loop (RCL) sequences are either API M358R (open box, with exploded sequence below) or M358R with additional F352A/L353V/E354V/A355I/I356A/I460L substitutions (RCL5, with open box inset “5”, and exploded sequence below). Recombinant proteins are identified to the right of each bar diagram. **Panel B** shows a reduced 12% SDS polyacrylamide gel stained with Coomassie Brilliant Blue. Ara-and Ara + lanes show total bacterial lysates from cultures expressing HV3API M358R grown in the presence or absence of 0.002% (w/vol) arabinose. Aliquots of bacterial lysates purified by nickel chelate chromatography (FT, flow-through; Ni, imidazole-eluted peak fractions; DEAE, final preparation following ion exchange on DEAE-Sepharose) are shown. **Panel C** shows two replicas of a reduced SDS gel loaded with 100 ng of each of the purified proteins identified above the lanes (GST; glutathione sulfotransferase, negative control) immunoblotted with either anti-API (upper panel) or anti-hexahistidine (anti-H6, lower panel). **Panel D** shows a reduced 12% SDS gel stained with Coomassie Brilliant Blue, containing 250 ng of each of the purified proteins identified above the lanes electrophoresed. The positions of molecular mass markers are labelled, in kDa, to the left of **Panels B-D**.

The HV3 peptides, HV3_54-66_, H_6_HV3_54-66_, and H_6_HV3_54-66_G_6_ (whose amino acid sequences are depicted in Figure 1, panel A) were produced by solid phase synthesis using 9-fluorenylmethyloxycarbonyl (Fmoc) chemistry, and purchased from the Advanced Protein Technology Center, The Hospital for Sick Children, (Toronto, ON).

### Construction of pBAD-H_6_HV3API M358R and pBAD-H_6_HV3API RCL5

All oligonucleotides used in this work were synthesized by the Institute for Molecular Biology and Biotechnology (MOBIX) at McMaster University (Hamilton, ON); the same facility provided DNA sequencing services used to confirm the bona fide nature of all candidate constructs. Previously described plasmids pBAD-H_6_HAPI M358R [28] and pBAD-H_6_HAPI RCL5 [24] were used as templates in PCR mediated by Phusion polymerase (New England Biolabs, Pickering, ON) in reactions primed by oligonucleotides 5′-CACCATGGGG TCTCATCATC ATCATCATCA TGGAGACTTT GAGCCTATCC CTGAGGATGC CTATGATGAG GGTGGAGGTG GAG GTGGAG AGGATCCCCA G-3′and 5′-CCGGAATTCT TATTTTTGGG TGGGATTCAC-3′, carried out under buffer and cycling conditions recommended by the enzyme manufacturer. The amplification products were restricted with Nco1 and EcoRI and the resulting 1271 bp fragments ligated between the corresponding sites in the plasmid pBADmychisB (Invitrogen, La Jolla, CA), producing novel plasmids pBAD-H_6_HV3API M358R and pBAD-H_6_HV3API RCL5, respectively. The open reading frame encoded by pBAD-H_6_HV3API M358R is depicted in Figure 1, Panel A, and comprised 421 codons specifying nonapeptide MGSH_6_, HV3 residues 54-66, spacer residues G_6_, and API M358R, in that order, from predicted N-terminus to C-terminus. The polypeptide encoded by pBAD-H_6_HV3API M358R was identical, except for the substitution of AVVIA for residues 352-356 inclusive (FLEAI) and of I360L, as previously described for API RCL5 [24].

### Construction of pBAD-H_6_API M358R-HV3 and pBAD-H_6_API M358R-G_6_HV3

In order to position HV3_54-66_ on the C-terminus of API M358R and API RCL5, an analogous PCR-mediated mutagenesis protocol was employed to that described above. Two constructs were made: one encoding API M358R with HV3_54-66_ fused directly to Lys394 (API M358R-HV3); and the second with a G_6_ spacer interposed between the API and HV3 components (API M358R-G_6_HV3). The entire open reading frame was PCR-amplified using sense oligonucleotide 5′-ATGCCATAGC ATTTTTATCC-3′ and antisense oligonucleotides 5′-TTCGAATTCT TACTCATCAT AGGCATCCTC AGGGATAGGC TCAAAGTCTC CTTTTTGGGT GGGATTCACC AC and 5′-TTCGAATTCT TACTCATCAT AGGCATCCTC AGGGATAGGC TCAAAGTCTC CTCCACCTCC ACCTCCACCT TTTTGGGTGG GATTCACCAC-3′, respectively. Resulting plasmid pBAD-H_6_API M358R-HV3 encoded the 407 amino acid polypeptide API M358R-HV3, while resulting plasmid pBAD-H_6_API M358R-G_6_HV3 encoded the 413 amino acid polypeptide API-M358R-G_6_HV3.

### Expression and purification of API M358R and related recombinant proteins

His-tagged recombinant proteins were purified from bacterial cell lysates prepared from arabinose-induced *E. coli* TOP10 cells transformed to ampicillin resistance by either previously described plasmid pBAD-H_6_HAPI M358R or pBAD-H_6_HAPI RCL5 or with one of the four novel plasmids described above. The same purification protocol was employed, utilizing Ni-NTA agarose (Qiagen) and DEAE-Sepharose (GE Healthcare, Baie d’Urfe, QC) chromatography of lysates from sonically disrupted bacteria as previously described [28], except that the elution fractions from nickel-chelate chromatography were dialyzed overnight in 20 mM sodium phosphate pH 6.8 prior to ion exchange chromatography on DEAE Sepharose (GE Health Care, Baie d’Urfe, QC), and a linear gradient of 0 to 300 nM NaCl in 20 mM sodium phosphate pH 6.8 was used to develop the DEAE Sepharose column. Fractions containing the protein of interest as determined by SDS-PAGE were pooled and concentrated using an Amicon Ultra-15 Centrifugal Filter Unit (EMD Millipore, Billerica, MA) and stored at -80°C. The concentrations of all recombinant serpin proteins were determined using spectrophotometry at OD280 nm, and calculated molar extinction coefficients based on their primary sequences, as described previously [40].

### Expression and purification of HCII 1-75

A small recombinant protein, 84 amino acids in size, comprising residues 1-75 of HCII with an N-terminal nonapeptide tag MSGH_6_ (designated HCII 1-75), was expressed in a pBAD-based plasmid and purified from sonicated cell lysates by nickel-chelate affinity chromatography as described [36].

### Thrombin clot time analysis

Thrombin clotting times were determined using an STA 4 coagulation analyzer (Diagnostica Stago, Asnieres sur Seine, France) and the Thrombin 10 reagent (Diagnostica Stago). Human citrated plasma was combined in a 1:3:5 volume ratio of plasma: veronal buffer: Thrombin 10 calcified thrombin reagent, and the time to clot was determined. The veronal buffer (sodium acetate trihydrate 7.14 mM/ sodium diethyl barbiturate 7.4 mM/ NaCl 0.131 M pH7.4) was employed with or without supplementation with purified recombinant serpins or synthetic peptides, as described [36].

### Competition of thrombin binding to immobilized HCII 1-75

A previously described assay was employed in order to characterize the relative affinity of recombinant serpins and peptides for either α-thrombin or α-thrombin rendered inactive at its active site by incubation with FPR-chloromethylketone [36]. Briefly, purified HCII 1-75 was immobilized on microtiter plates and purified human α-thrombin was incubated with or without competitor peptides or proteins. Thrombin binding, following washes, was detected with a horseradish peroxidase-conjugated sheep anti-human affinity-purified antibody (Affinity Biologicals, Ancaster, ON), binding isotherms were solved for one-site binding by non-linear regression, and the concentration of competitor needed to reduce the binding by 50% (IC50) was calculated as described [36].

### Gel based serpin enzyme complex analysis

The ability of HV3API M358R and API M358R to form SDS-stable complexes with α-thrombin was measured by incubating 1 μM serpin with 0.1 μM α-thrombin at ambient temperature at various time points over 3 minutes. Reactions were stopped with SDS and analysed on 10% SDS-PAGE gels as previously described [28,41].

### Kinetic analysis of α_1_-PI variants and fusion proteins

The apparent second-order rate constant (k_2_) of inhibition of 10 nM α-thrombin or γ_T_-thrombin by recombinant serpins was determined under pseudo-first order conditions involving a 10-to 65-fold molar excess of serpin over protease in the first stage of a two-step discontinuous assay, as previously described [28,42]. In the second stage, reactions were diluted into 100 μM chromogenic substrate S2238 (DiaPharma,West Chester OH) and the change in absorbance at 405 nm was followed for 5 minutes in an ELx808 plate reader (BioTek Instruments, Winooski, VT). The same approach was used to measure the rate of inhibition of 10 nM APC by recombinant serpins, except that a 1000-fold excess of serpin over protease was employed, and 400 μM chromogenic substrate S2366 (DiaPharma) was employed in the second stage. In addition, stoichiometries of α-thrombin inhibition were determined by incubating recombinant serpins (0-800 nM) with 200 nM thrombin for two hours at room temperature. This reaction was diluted into 150 μM chromogenic substrate S2238 and residual thrombin activity was measured as described above. The number of serpin molecules required to inhibit one molecule of thrombin was calculated by plotting the residual thrombin activity versus the ratio of serpin to thrombin and extrapolating to complete inhibition (zero thrombin activity) by linear regression [25,28].

### Statistical analysis

Data analysis was performed using computer software (InStat, Version 3.06, GraphPad Software, San Diego, CA, USA); graphs were also produced with using software from the same company (GraphPad Prism, Version 4.03). Comparisons were made between parent proteins and modified proteins (e.g. API M358R with HV3API M358R or API RCL5 versus HV3API RCL5) unless otherwise stated, using parametric tests (unpaired two-tailed t tests) if they passed conditions of normal distribution and similarity of standard deviations, or non-parametric tests (Mann–Whitney) if they did not meet these conditions. A p value > 0.05 was considered not significant in all cases.

## Results

### Recombinant protein design and expression

As shown in Figure 1, Panel A, as a first step in this project, recombinant serpins were designed for expression in *E. coli*. The same general strategy was employed as in previous reports, in which the N-terminal extension of HCII had been grafted onto either singly mutated API M358R, or multiply mutated API RCL5 (API M358R with six additional RCL substitutions), except that HV3 residues 54-66 was separately exchanged for HCII 1-75, in both cases [24,29]. Induction of bacterial cultures harbouring expression plasmids specifying HV3API M358R with arabinose led to the appearance of a novel 47 kDa protein in total soluble lysate preparations, that was enriched by nickel-chelate chromatography and purified following an additional ion exchange step, like API M358R and HAPI M358R (see Figure 1, Panel B). The putative HV3API polypeptide reacted both with antibodies specific to API and to hexahistidine (see Figure 1, Panel C), and exhibited a mobility intermediate between API M358R and HAPI M358R, consistent with its theoretical molecule mass of 47,356 Da (see Figure 1, Panel D). Taken together, these characteristics identified the candidate protein as HV3API M358R; similar results were obtained for HV3API RCL5 (see Figure 1, Panel C).

### Initial electrophoretic characterization of HV3API M358R

**Figure 2:**
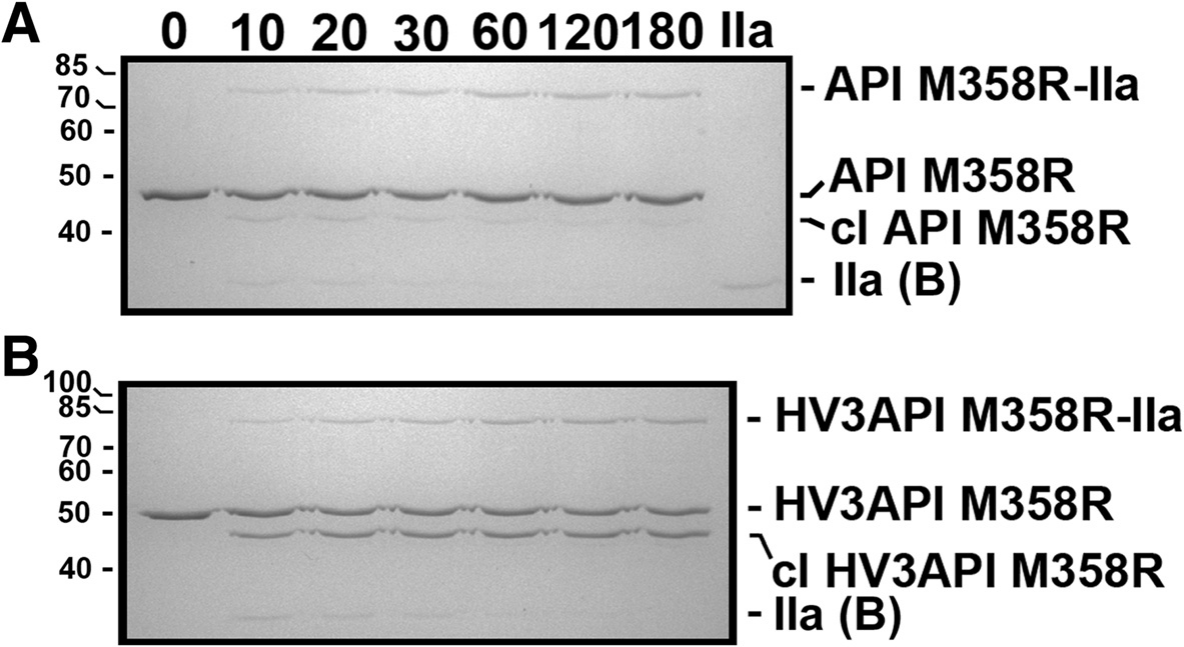
**Formation of SDS-stable complexes between fusion proteins and thrombin.** Either 1.0 μM API M358R **(Panel A)** or 1.0 μM HV3API M358R **(Panel B)** was combined with 0.2 μM thrombin (IIa) for the time in seconds shown above the lanes. Reactions were stopped with SDS and aliquots were electrophoresed on reduced 10% SDS polyacrylamide gels, and stained with Coomassie Brilliant Blue. Thrombin-dependent serpin-enzyme complexes with retarded mobility (either API M358R-IIa or HV3API M358R-IIa), unreacted serpins, cleaved serpins (cl API M358R or cl HV3API M358R), or the B chain of thrombin (IIa (B)) are labelled, at right. The position of molecular mass markers is shown, in kDa, to the left of each panel.

The ability of HV3API M358R to inhibit thrombin, and form denaturation-resistant inhibitory complexes with thrombin, was initially assessed electrophoretically under reducing conditions. As shown in Figure 2, Panel A, when excess purified API M358R (1.0 μM) was combined with thrombin (100 nM), a 76 kDa API M358R-IIa complex was detectable by 10 seconds elapsed time, increasing in intensity up to 120-180 seconds, with concomitant disappearance of the thrombin (visualized as the B chain under reducing conditions). Qualitatively indistinguishable results were obtained when HV3API M358R was reacted with thrombin under identical conditions (see Figure 2, Panel B), except that the serpin-enzyme complex displayed slightly reduced mobility consistent with the larger mass of HV3API M358R (78 kDa) and the formation of a cleaved form HV3 API M358R with slightly more rapid mobility than the intact serpin was more pronounced compared to API M358R. Similar results were obtained with HV3API RCL5 (data not shown).

### Kinetic characterization of HV3API M358R and HV3API RCL5

The rate of reaction of the two API and their corresponding HV3API variant proteins with α-thrombin, γ_T_-thrombin, and APC was quantified by determining the second order rate constant of inhibition (k_2_) for each protease. As shown in Table 1, fusing HV3 residues 54 to 66 was associated with a statistically significant, 3.3-fold increase in the mean k_2_ for inhibition of α-thrombin by API M358R (p = 0.0022 by Mann–Whitney test). Similarly, grafting the same residues from HV3 onto API RCL5 was associated with a statistically significant, 1.9-fold increase in the mean k_2_ for inhibition of α-thrombin by API RCL5 (p < 0.0001 by unpaired t test). The mean k_2_ of HV3API M358R for thrombin inhibition, although elevated relative to API M358R remained 2.6-fold lower than HAPI M358R, resulting in an order of potency as thrombin inhibitors of API M358R < HV3API M358R < HAPI M358R. Substituting γ_T_-thrombin, a proteolytic derivative of intact α-thrombin possessing only a vestigial exosite 1 [33], for the intact protease, reduced the enhancement associated with fusion of the HV3 residues to 1.8-fold in the case of HV3API M358R, and 1.4-fold in the case of HV3API RCL5, although the differences remained statistically significant, and the order of potency remained unchanged to that observed with α-thrombin.

**Table 1:** **Second order rate constants (**k_2_**) of inhibition for recombinant serpins**

	**Serpin with M358R loop**			**Serpin with RCL5 loop**	
**Protease**	**API M358R**	**HV3API**	**HAPI M358R**	**API RCL5**	**HV3 API RCL5**
		**M358R**			
α-thrombin (×10^6^ M^-1^ min^-1^)	22 ± 4	72 ± 15***	186 ± 32***	35 ± 8	66 ± 8***
γ_T_-thrombin (×10^6^ M^-1^ min^-1^)	5.9 ± 0.1	11 ± 3**	34 ± 10**	15 ± 0.8	21 ± 3***
APC (×10^6^ M^-1^ min^-1^)	1.7 ± 0.1	1.9 ± 0.2	1.7 ± 0.2	0.0038 ± 0.0013^a^	0.022 ± 0.005***
α-thrombin/ APC ratio	13	39	109	9,211	3000

Kinetic analysis of protease inhibition by API fusion proteins and variants. The mean of at least 5 k_2_ determinations ± SD is shown (×10^6^ M^-1^ min^-1^). ^a^Value from Sutherland et al., 2007. Values that are statistically significantly different from parent protein values (HV3API M358R and HAPI M358R compared to API M358R, HV3API RCL5 compared to API RCL5), **, p <0.01, ***, p < 0.001 by Mann–Whitney or two-tailed t test. Further details are provided in the text.

With respect to the ability of the recombinant serpins to inhibit APC, no difference was found between the k_2_ determined with API M358R and that found with HV3API M358R; in contrast the addition of HV3 residues 54-66 was associated with a 5.8-fold increase in the mean k_2_ observed with unfused API RCL5 (p < 0.001 by unpaired t test). In terms of the relative specificity of the recombinant serpins for α-thrombin versus APC, the kinetic results led to a ranking of API M358R = HV3API M358R = HAPI M358R > HV3API RCL 5 > API RCL5 (see Table 1). The stoichiometry of thrombin inhibition (SI) was tested for API M358R, HAPI M358R, and HV3API M358R; both fusion proteins exhibited a statistically significant increase in SI, from 2.0 ± 0.3 for API M358R to 2.4 ± 0.2 for HAPI M358R to 3.3 ± 0.1 for HV3API M358R. The stoichiometry of APC inhibition was not determined.

### Comparison of HV3 peptides to HCII 1-75

**Figure 3:**
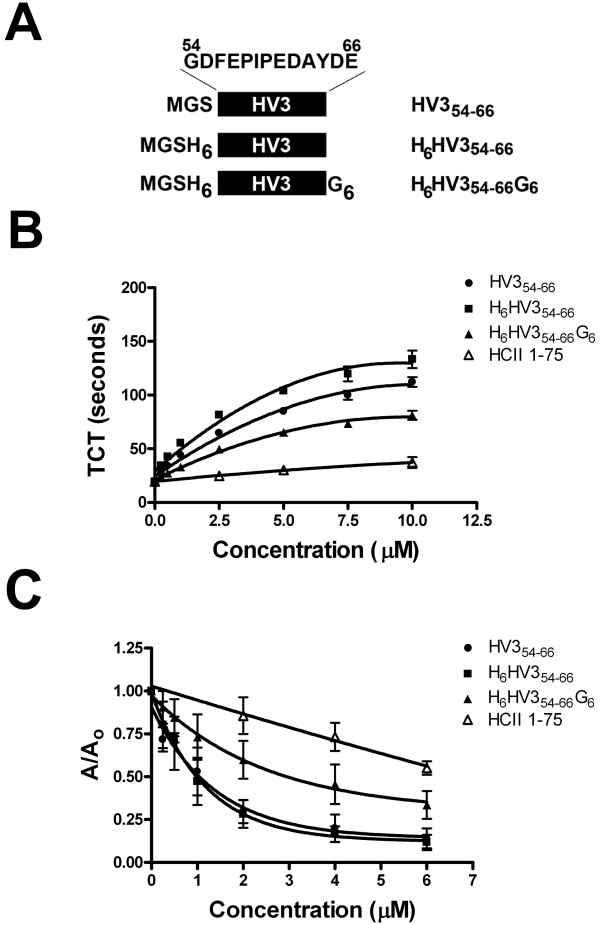
**Inhibition of thrombin clotting time and HCII 1-75-thrombin binding assays by HV3 peptides and HCII 1-75. Panel A** depicts three synthetic peptides schematically. All contain HV3 residues 54-66 (sequence shown exploded, above first HV3_54-66_ bar), shown as a black bar (with “HV3” inset in white) with an MGS tripeptide on the N-terminus, without (HV3_54-66_) hexahistidine or hexaglycine components or with only hexahistidine (H_6_HV3_54-66_) or with both hexahistidine and hexaglycine components (H_6_HV3_54-66_G_6_). **Panel B** shows the Thrombin Clotting Time (TCT) in seconds; diluted citrated human plasma was recalcified in the presence of thrombin and increasing concentrations of competitor peptides identified at right. The mean ± SD of 3 to 4 determinations is shown. **Panel C** shows binding of α-thrombin to immobilized HCII 1–75, expressed as the ratio of the absorbance at 490 nm in the presence of competitor **(A)** to that in its absence (A_0_); competitor peptides are identified at right. The mean ± SD of 5 to 6 determinations is shown.

Synthetic peptides containing HV3 residues 54-66 were next compared to HCII 1-75, with respect to anticoagulant properties and their relative abilities to bind to α-thrombin. As shown in Figure 3, Panel A, three HV3-related peptides were used: HV3_54-66_, comprising only those residues plus an MGS tripeptide on the N-terminus; H_6_HV3_54-66_, with a hexahistidine tag between MGS and HV3 residues; H_6_HV3_54-66_G_6_, with both a hexahistidine tag and a hexaglycine spacer. As shown in Figure 3, Panel B, all three HV3-related peptides had greater anticoagulant activity than HCII 1-75, when added into Thrombin Clotting Time (TCT) assays at increasing concentrations in the μM range. The order of inhibitory potency was found to be: HV3_54-66_ > H_6_HV3_54-66_ > H_6_HV3_54-66_G_6_ > HCII 1-75. The concentrations of these inhibitors required to double the TCT were, respectively (mean, in μM, ± SD): 0.45 ± 0.01; 0.93 ± 0.05; 1.6 ± 0.1; and 12 ± 4. The HV3-related peptides, by this measure, were therefore 8-to 27-fold more potent inhibitors of clotting than HCII 1-75.

Similar results were obtained when the HV3-related peptides and HCII 1-75 were used as competitors of the binding of thrombin to immobilized HCII 1-75, in a previously described binding assay [36] shown in Figure 3, Panel C. In this assay, the order of inhibitory potency was found to be: HV3_54-66_ = H_6_HV3_54-66_ > H_6_HV3_54-66_G_6_ > HCII 1-75. This order was reflected in IC_50_ values derived from the inhibitory binding isotherms (mean, in μM, ± SD) of: 0.90 ± 0.3; 0.74 ± 0.2; 1.4 ± 0.3; and 8.0 ± 1. The differences in IC_50_ values among HV3-related peptides were not statistically significant, but each HV3-related peptide exhibited a significantly lower IC_50_ than that of HCII 1-75 (p < 0.001 by ANOVA with Tukey-Kramer post-tests).

### Competition of binding of HCII 1-75 to active site-inhibited thrombin

**Figure 4:**
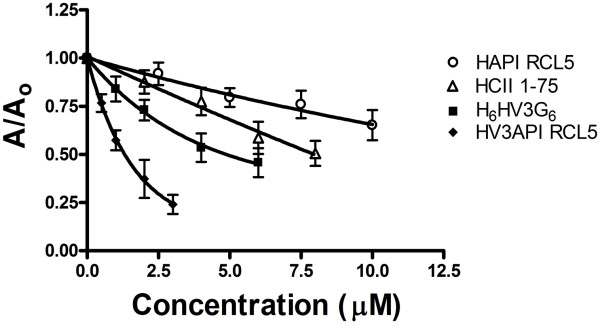
**Competition of binding of FPR-ck-thrombin to HCII 1-75 immobilized on microtiter plate wells.** Binding of FPR-ck-thrombin, expressed as the ratio of the absorbance at 490 nm, in the presence (A) of competitor peptides (HCII 1-75 or H_6_HV3G_6_) or competitor proteins (HAPI RCL5 or HV3API RCL5), to that in its absence (A_0_), is shown. Results are the mean ± SD of five determinations.

In order to extend the competitive binding experiments to include N-terminally extended serpin fusion proteins, focusing only on their ability to interact with thrombin via exosite 1, and not via thrombin’s active site, we substituted active site-inhibited FPR-ck thrombin for α-thrombin and used peptides and fusion proteins to inhibit FPR-ck thrombin binding to immobilized HCII 1-75. As shown in Figure 4, HCII 1-75 was a more effective competitor in free form than when fused to API RCL5, while H_6_HV3_54-66_G_6_ was more effective when fused to API RCL5 than when present in free form. This qualitative assessment of the binding isotherm was borne out by quantification using IC_50_ values, which were determined to be (mean, in μM, ± SD): H_6_HV3_54-66_G_6_, 5 ±1; HV3API RCL5, 1.4 ± 0.3; HCII 1-75, 9 ± 2; HAPI RCL5, 17 ± 2. The differences between unfused extensions and corresponding fusion proteins in IC_50_ values were statistically significant (i.e. H_6_HV3_54-66_G_6_ versus HV3API RCL5, p = 0.002 by unpaired t test, and HCII 1-75 versus HAPI RCL5, p = 0.003 by unpaired t test) but qualitatively reversed, in that HCII 1-75 was a more effective competitor of FPR-ck thrombin binding in free form while H_6_HV3_54-66_G_6_ was a more effective competitor when present in fused form. Overall, HV3API RCL5 was also a better ligand for thrombin exosite 1 than HAPI RCL5.

### Gel-based and kinetic activity assays of C-terminal HV3 fusions to API M358R

**Figure 5:**
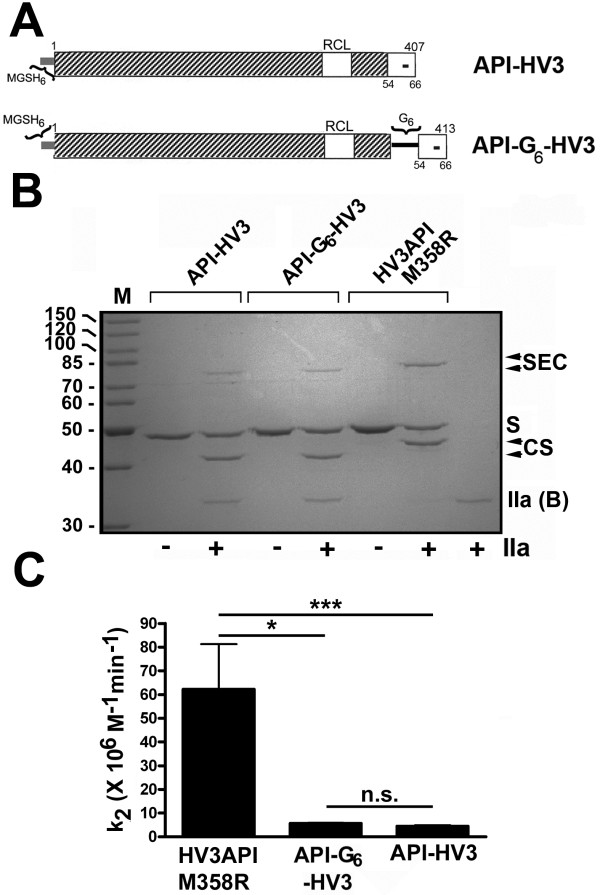
**Formation of SDS-stable complexes between thrombin and N-terminal or C-terminal HV3 54-66–API M358R fusion proteins.** Polypeptides comprising portions of API M358R (hatched bar, with residue numbers above) with or without C-terminal extensions (white) derived from HV3 (with single “minus sign” symbols inset, and residue numbers below) are represented schematically in **Panel A**. Each protein contains an N-terminal MGSH6 tag (shown at left of each schematic representation as a thin grey bar). Fusion proteins contain (API-G_6_-HV3) or do not contain (API-HV3) a six residue spacer peptide (thin black bar) between C-terminal extension and API M358R body identified above the bar. Reactive centre loop (RCL) sequences are API M358R (open box) in both cases. **Panel B** shows a reduced 10% SDS polyacrylamide gel stained with Coomassie Brilliant Blue. Proteins identified above the lanes were incubated (1.0 μM serpin, 0.2 μM thrombin) without (-) or with (+) thrombin (IIa) at ambient temperature for 1.0 minute. The position of the serpin-enzyme complex (SEC) is highlighted with arrowheads. The position of unreacted serpin (S), cleaved serpin (CS) and the B chain of thrombin (IIa (B)) is shown at right. M indicates molecular mass markers of 160, 120, 100, 90, 80, 70, 60, 50, 40, and 30 kDa, respectively. **Panel C** shows a plot of the second order rate constants of thrombin inhibition (k_2_, in units of 10^6^ M^-1^ min^-1^) of the purified proteins identified, in each case, below the x axis. The mean ± SD for 9, 6, and 5 replicated determinations is shown. Lines between the columns indicate statistical significance (non-parametric ANOVA, Kruskal-Wallis test with Dunn’s post-tests): *, p < 0.05; ***, p < 0.001; n.s., not significant).

Two additional fusion proteins were designed, expressed, and purified, using the same arabinose-inducible bacterial expression system employed throughout this study, in order to determine if appending the HV3 triskaidecapeptide to the C-terminus of API M358R, a location corresponding to its natural position on the C-terminus of HV3, would yield a more active thrombin-inhibitory serpin. As shown in Figure 5, Panel A, API-HV3 was designed to be identical to API M358R except for the C-terminal addition of HV3 54-66; API-G_6_-HV3, in turn, was identical to API-HV3 except for the positioning of a G_6_ spacer between API Lys394 and HV3 Glu66. Purified API-G_6_-HV3 co-migrated with purified HV3 API M358R, as expected given their identical amino acid composition, but different amino acid sequence, while purified API-HV3 migrated slightly more rapidly than the other two recombinant proteins, consistent with its lacking the hexaglycine sequence present in the other two (see Figure 5, Panel B, “-IIa” lanes). When all three proteins were separately incubated in identical excess of α-thrombin, less serpin-enzyme complex appeared to form in a one minute reaction in the case of the C-terminal HV3 fusions than was observed with HV3API M358R (see Figure 5, Panel B). Densitometry of the stained gel supported this visual impression, and showed that while 37% of initial HV3API M358R was converted into a covalent complex with thrombin, only 15% of API-G_6_-HV3 and 32% of API-HV3 reacted in the same manner. The rates of reaction of these three proteins were further compared by kinetic analysis (see Figure 5, Panel C). The second order rate constant of inhibition (k_2_) for the three inhibitors was found to be 60 ± 20 for HV3API, 5.7 ± 0.3 for API-G_6_-HV3, and 4.5 ± 0.4 for API-HV3 (all ×10^6^ M^-1^ min^-1^, mean ± SD of 9, 6, and 5 determinations). These 11-and 14-fold reductions in the rate of thrombin inhibition by the API variants with C-terminal fusions of the HV3 C-terminal triskaidecapeptides were statistically significant (p < 0.05 and p <0.001 versus HV3API M358R value; see Figure 5C).

## Discussion

In this study we sought to enhance the inhibitory potency of API M358R, a variant form of API that inhibits thrombin. Our primary hypothesis was that fusion of the C-terminal triskaidecapeptide of hirudin to the N-terminus of API M358R would increase the rate of reaction of the resulting novel engineered serpin with thrombin. Our reasoning was based on our previous demonstration that fusion of the N-terminal 75 amino acids of HCII increased the reactivity of API M358R and other forms of API with thrombin; both HCII 1-75 and the C-terminal region of hirudin are known to interact with thrombin exosite 1. Deletion of HCII 1-75 in HCII reduced the rate of thrombin inhibition of recombinant HCII [32,34,43,44] and adding HCII 1-75 to API M358R increased it [28]. Our secondary hypothesis was that fusing the exosite 1-binding motif from hirudin to API M358R or RCL5 would accelerate the rate of thrombin inhibition by API M358R to a greater extent than fusing HCII 1-75. Our reasoning here was based on observations that hirudin C-terminal peptides bound thrombin with higher affinity than that exhibited by HCII 1-75 or relative peptides [36,37].

Our results supported our first hypothesis. Fusing HV3 residues 54-66 to either API M358R or API RCL significantly increased the rate of thrombin inhibition of these recombinant serpins, by approximately 3-fold. The effect was reduced when γ_T_-thrombin was substituted for α-thrombin, consistent with the enhancement being tied to the presence of a fully functional exosite 1; this exosite is largely disrupted by trypsin-mediated proteolysis in γ_T_-thrombin [33]. The enhancement was thrombin-specific, in that no change in APC inhibition rate was found between API M358R and HV3API M358R. While it is true that HV3AP1 RCL5 exhibited a 5-fold more rapid rate of APC inhibition than API RCL5, both proteins were poor inhibitors of APC, with the reduction in APC inhibition rate being derived primarily from the antithrombin-like RCL found in the RCL5 proteins; a similar phenomenon was previously noted for fusion protein HAPI M358R, likely indicative of overall stabilization of the fusion protein, since APC contains no known analogues of thrombin exosite 1 [28]. As expected based on the precedent of altering HAPI M358R to HAPI RCL5, there was no further increase in the rate of thrombin inhibition gained by altering HV3API M358R to HV3API RCL5, but instead a considerable increase in specificity attributable to the decreased ability of API M358R variants bearing this more antithrombin-like RCL to inhibit APC [24,26].

Serpins follow a branched reaction pathway with proteases, acting either as inhibitors or substrates. We previously reported the ratio between substrate and inhibitor outcomes, the stoichiometry of inhibition, as being similar between API M358R and HAPI M358R, of 2.9 to 3.2. In the current study we found that API M358R had the least tendency to act as a substrate, with an SI of 2.0, while the acquisition of a faster rate of inhibition of HAPI M358R (mean SI 2.4) and in particular HV3API M358R (mean SI 3.3), had been achieved at the cost of greater substrate-like behavior.

In contrast to the general findings discussed above that supported our primary hypothesis, our second hypothesis was not supported by our results. We first followed thrombin-serpin complex formation using gel-based assays. While these experiments revealed no unusual complex instability of the novel variant serpins, as has been previously reported for some variants [42,45-47], they also showed no discernible difference in the rate of thrombin inhibition between API M358R and the HV3-containing fusion proteins. This finding suggested that the rate enhancement was likely less than 5-to 10-fold, given our previously demonstrated ability to note a rate enhancement with the relatively insensitive electrophoretic assay in this range of difference [24,29]. This prediction was borne out in the kinetic analysis. HV3API M358R exhibited a mean k_2_ of α-thrombin inhibition of 7.2 × 10^7^ M^-1^ min^-1^, one clearly inferior to rate constants we reported for HAPI M358R of 1.4 to 2.3 × 10^8^ M^-1^ min^-1^[28,29].

To explain why we had failed to capitalize on the greater affinity of the hirudin C-terminal motif than HCII 1-75 for thrombin exosite 1 in HV3API M358R, we considered the possibility that our selection of the C-terminal residues of hirudin variant 3, rather than other variants of hirudin, was to blame. This explanation was eliminated by our finding that synthetic peptides comprising HV3 54-66, with or without the hexaglycine or hexahistidine motifs present in HV3API M358R, inhibited thrombin-mediated clotting assays and competed for the binding of thrombin to immobilized HCII 1-75 more effectively than free HCII 1-75. The latter finding was consistent with our previous demonstration of a ten-fold advantage of a hirudin variant 1 55-65 peptide over HCII 1-75 as an exosite 1 ligand, deduced using surface plasmon resonance [36]. Substituting FPR-ck-thrombin for α-thrombin in the immobilized HCII 1-75 binding assay next permitted comparisons of the free and fused versions of the HV3 and HCII 1-75 exosite 1-binding motifs to be made. HCII 1-75 bound thrombin exosite 1 better in free form than in the context of the HAPI RCL5 fusion protein, while HV3 54-66 in the HV3API RCL5 context was a superior ligand not only in comparison to either free or fused HCII 1-75 but also in comparison to its unfused form. This advantage in binding thrombin exosite 1 was not translated into superiority as a serpin; HV3API RCL5 exhibited a mean k_2_ for α-thrombin inhibition of 6.6 × 10^7^ M^-1^ min-^1^ compared to 1.4 × 10^8^ M^-1^ min^-1^ for HAPI RCL5.

The differences uncovered between the ability of the fusion proteins to bind thrombin exosite 1 and the ability to form a serpin-enzyme complex with thrombin suggested that the higher affinity HV3 motif might recruit α-thrombin to HV3-API proteins more effectively than to HCII 1-75-API proteins, but orient them in a less favourable way for attack by the thrombin active site on the RCL. Noting that the exosite 1-binding motif in hirudin is found on the C-terminus of the protein, we switched the fusion point of HV3 54-66 from the N-terminus of API M358R to the C-terminus of the serpin. While functional, the resulting fusion protein, with or without a spacer peptide, inhibited thrombin at rates 11-to 14-fold less than those achieved by HV3API M358R. C-terminal extensions are known within the serpin family in spite of the proximity of this region to the RCL, most notably in alpha-2-antiplasmin [1], and a C-terminal hexahistidine extension conferred enhanced thrombin inhibitory activity on HCII in the presence of heparin [43]. In spite of these precedents, positioning the exosite 1-binding motif of hirudin in this location did not lead to enhanced function of API M358R. This inferiority may have arisen for steric or conformational reasons; the HV3 motif may either have been too close to the RCL or inappropriately angled to permit simultaneous productive engagement of both exosite 1 and the active site of thrombin. Variant forms of API M358R with attached HV3 triskaidecapeptides, in either location, could bind thrombin via exosite-1; indeed, we demonstrated this property of HV3API M358R using FPR-ck-thrombin in this report. It is possible that any of the HV3-API fusion proteins could bind thrombin exosite 1 and interfere with the subsequent attack of another HV3-API fusion protein on the RCL. Similarly, cleavage of the RCL in API-HV3 or API-G_6_-HV3 by thrombin would liberate a small polypeptide chain commencing with P1′ and terminating with the C-terminus of the fusion proteins, including the HV3 triskaidecapeptide. Competition of these small polypeptides for exosite-1 might then have some inhibitory effects, although it should be noted that we previously found that a 1000-fold molar excess of HCII 1-75 peptide only reduced the rate of thrombin inhibition by glycosaminoglycan-catalyzed HCII by approximately 4-fold [36].

**Figure 6:**
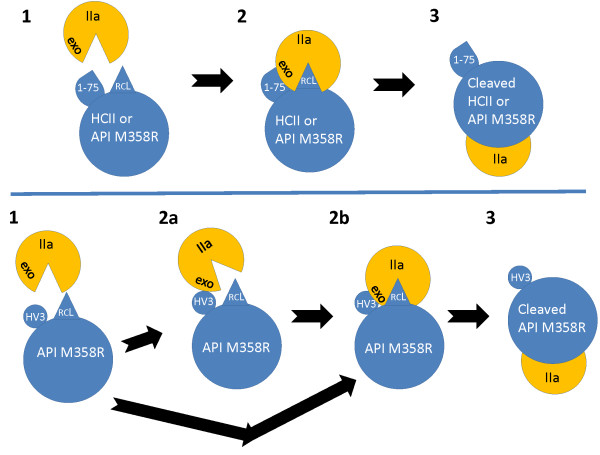
**Schematic comparison of reactions of thrombin with natural and engineered serpins.** The upper panel depicts the reaction of thrombin (yellow) with heparin-activated HCII or HCII-API fusion protein HAPI M358R (blue). **(1)** Thrombin (IIa, with pie-shaped active site canyon opening and labelled exosite 1 (exo) encounters heparin-activated HCII or fusion protein HAPI-M358R, both containing HCII 1-75 (1-75, blue teardrop-shaped extension) and a Reactive Centre Loop (RCL, blue triangle) on a serpin scaffold (blue large oval). Interactions between HCII 1-75 and exosite 1 and the serpin RCL with thrombin’s active site guide the serpin into productive encounter complex formation **(2)**. Aligned encounter complex formation leads to final serpin-enzyme complex formation, with translocation of thrombin; the RCL is no longer visible due to its conversion into a β-strand inserted into the body of the serpin **(3)**. The lower panel depicts the reaction of thrombin (yellow) with HV3API M358R (blue), in which the C-terminal triskaidecapeptide of HV3 (blue circular extension) was substituted for HCII 1-75. For this fusion protein, initial complexes form which comprise either a sub-optimally aligned complex in which exosite 1 and HV3 interact without engagement of the RCL with thrombin’s active site **(2a)**, or the appropriately aligned encounter complex forms directly **(2b)**. Suboptimally aligned complexes from **(2a)** can proceed to optimal alignment **(2b)** but misalignment is predicted to be more likely with HV3API M358R than with HAPI M358R or heparin-activated HCII. Apart from the greater size of the serpins with respect to thrombin, diagrams are not to scale.

The similarity between the acidic extension of HCII and the C-terminal motif in hirudin has long been known [32,48]. When an encounter complex structure between thrombin S195A and HCII was solved [31], it became possible to compare the interactions between the partially resolved HCII extension and thrombin exosite 1 and the corresponding, fully resolved interactions in hirudin variants 1 and 2 [49,50]. In spite of the enrichment in acidic residues in both motifs, it was hydrophobic residues, in helical conformation in HCII, and in a non-helical conformation in hirudin, that formed the closest contacts with the hydrophobic face of exosite 1[31]. The residues in hirudin (HV3 numbering) include F56, I59, P60, and Y64, and in HCII analogous residues L61, L63, I66, F67, and I74 [31]. In spite of our efforts to position HV3 54-66 in an orientation in HV3API M358R as close as possible to the corresponding exosite 1-binding residues in HAPI M358R, it would appear that the conformation of the resulting fusion protein was sub-optimal with respect to the two goals of binding exosite 1 and simultaneously positioning the RCL for efficient attack by the active site of thrombin (see Figure 6). In native HCII, glycosaminoglycan binding is necessary, allosterically activating the serpin, to expel its RCL from a partially inserted position in β-sheet A and stretch it to an extent sufficient both to bind thrombin’s active site and to permit acidic extension-exosite 1 interaction [31]. In HCII-API M358R fusion proteins, some aspects of this optimal structure were recreated on a serpin scaffold of similar size and conformation to glycosaminoglycan-activated HCII. Our results in this study suggest that further enhancements to the thrombin inhibitory capacity of API M358R and related variants should be built on this scaffold. For instance, one or both acidic repeats (including related hydrophobic areas of HCII) could be replaced with the corresponding motif from HV3, in the context of the full HCII 1-75 extension. At least in the context of API M358R fusion proteins [28] or isolated HCII extensions [36], we have shown that the full extension is required for maximal interaction with thrombin. In this regard it has also been noted that the triple mutation D72N/Y73F/D75 confers a 4-to 6-fold increased rated of thrombin inhibition on recombinant HCII in either the absence or presence of glycosaminoglycans [35], and this alteration could also form part of future API engineering efforts to increase the potency of API as a potential antithrombotic agent.

## Conclusions

In this study we have demonstrated that enhancements in the rate of thrombin inhibition by API M358R and related variants can be achieved by fusing a thrombin exosite 1-binding motif on the N-terminus of the recombinant serpin. This approach increased the rate of inhibition of α-thrombin inhibition both for HV3API M358R, and also for HV3API RCL5, in which six RCL residues in addition to M358R were mutated to the corresponding residues in antithrombin. Full enhancement was dependent on the integrity of exosite 1 in thrombin. Although the approach was successful in increasing the rate of thrombin inhibition, fusion proteins containing the acidic extension of HCII, such as HAPI M358R and HAPI RCL5, were faster thrombin inhibitors. Both in isolated and fused form, HV3 54-66 bound thrombin exosite 1 more tightly than HCII 1-75; however, this tight binding did not lead to enhanced rates of thrombin inhibition, suggesting that it may have locked the recombinant serpins into exosite 1-bound states that were not optimal for subsequent serpin-enzyme complex formation. Future protein engineering of thrombin inhibitors based on API M358R could more productively include the high affinity of the hirudin C-terminal region if the analogous residues were presented in the context of a mutated HCII 1-75 fusion moiety.

## Abbreviations

API: α_1_-proteinase inhibitor, α_1_-antitrypsin; API M358R: API with the substitution of Met358 by Arg; γT-thrombin: Proteolytic fragment of α-thrombin formed by digestion with trypsin; BSA: Bovine serum albumin; FPR-ck: D-Phe-L-Pro-L-Arg chloromethylketone, active site inhibitor of thrombin; HAPI: Fusion protein of residues 1-75 of heparin cofactor II and all of α_1_-PI; HAPI M358R: HAPI with the M358R substitution; HAPI RCL5: HAPI M358R with additional F352A/L353V/E354V/A355I/I356A/I460L substitutions; HCII: Heparin cofactor II; HCII 1-75: Recombinant peptide containing the first 75 residues of human HCII and a nonapeptide N-terminal tag; HV3: Hirudin variant 3; HV3 54-66: N-acetylated synthetic peptide containing the C-terminal 13 residues of HV3; HV3API M358R: Fusion protein of residues 54-66 of HV3, preceded by MGSH_6_, followed immediately by G_6_, and then by API M358R, in N-to C-terminal order; k2: Second order rate constant of inhibition; RCL: Reactive centre loop; serpin: Serine protease inhibitor; P1-P1′: The reactive centre peptide bond, where P1 is the amino acid N-terminal to cleavage and P1′ is the amino acid C-terminal to cleavage; SDS: Sodium dodecyl sulfate; SDS-PAGE: SDS polyacrylamide gel electrophoresis; WT: Wild-type.

## Competing interests

The authors declare that they have no competing interests.

## Authors’ contributions

WPS conceived of the study, secured competitive funding, directed experiments, and edited and revised the manuscript. LAR and VB performed all experiments and developed and refined experimental protocols. LAR wrote the first draft of the manuscript, which was derived from experiments contained within her M. Sc. Thesis (Medical Sciences, McMaster University). All authors participated in editing and revising the manuscript. All authors read and approved the final manuscript.
